# Pretreatment microRNA levels can predict HBsAg clearance in CHB patients treated with pegylated interferon α-2a

**DOI:** 10.1186/s12985-018-0982-y

**Published:** 2018-04-23

**Authors:** Yanlin Yang, Ming Liu, Ying Deng, Yan Guo, Xuqing Zhang, Dedong Xiang, Li Jiang, Zhonglan You, Yi Wu, Maoshi Li, Qing Mao

**Affiliations:** 1 Department of Infectious Diseases, Southwest Hospital, Third Military Medical University (Army Medical University), Chongqing, 400038 China; 2 The Chongqing Key Laboratory for Research of Infectious Diseases, Chongqing, 400038 China

**Keywords:** Peripheral blood mononuclear cells, microRNA, HBsAg clearance, Pegylated interferon α-2a

## Abstract

**Background:**

To investigate the predictive capability of microRNAs (miRNAs) prior treatment for HBsAg clearance in chronic hepatitis B (CHB) treated with pegylated interferon α-2a (PEG-IFNα-2a).

**Methods:**

The treatment effect was determined by HBsAg clearance and subjects were classified into HBsAg clearance group and non HBsAg clearance group. Differential miRNAs expression in peripheral blood mononuclear cells (PBMC) was screened using microarrays in an identification cohort (*n* = 20) and validated by quantitative reverse-transcription polymerase chain reaction (qRT-PCR) in a confirmation cohort (*n* = 47). Receiver operating characteristic curve (ROC), logistic regression and gene ontology (GO)/Pathway analyses were used to evaluate the predictive capability of selected miRNAs for HBsAg clearance and determine their mechanistic roles.

**Results:**

Twenty-seven subjects (40.3%) acquired HBsAg clearance, ten in the identification cohort and seventeen in the confirmation cohort. Four miRNAs out of twelve (miR-3960, miR-126-3p, miR-335-5p, miR-23a-3p) were verified to be differential expressed by qRT-PCR in the confirmation cohort. Their expression patterns were consistent with the microarray results. Their levels were lower in the response group compared with the nonresponse group (*p* < 0.05). The areas under curve (AUC) were 0.8333 (*p* = 0.001), 0.751 (*p* = 0.01), 0.7294 (*p* = 0.013), 0.6275 (*p* = 0.094) and positive predict values (PPV) were 84.62, 60.00, 70.00, 28.57% for miR-3960, miR-126-3p, miR-335-5p, and miR-23a-3p respectively. The AUC and PPV of the combination of miR-3960 and miR-126-3p were 0.8529 and 92.31%, which were better than using miR-3960 alone, but the differences were not statistically significance (*p* > 0.05).

**Conclusions:**

We identified differential expressed miRNAs between response and nonresponse groups of PEG-IFNα-2a treatment and demonstrated that miR-3960 was the optimal predictor for HBsAg clearance compared with other miRNAs, but it requires to be further comfired in larger cohort studies.

**Trial registration:**

ChiCTR ChiCTR-ROC-16008735, registered retrospectively on 28 June, 2016.

**Electronic supplementary material:**

The online version of this article (10.1186/s12985-018-0982-y) contains supplementary material, which is available to authorized users.

## Background

Chronic hepatitis B virus infection is a global health problem harming human health. Approximately 240 millions are chronic HBsAg carriers and about one million people die annually from HBV associated diseases [1, 2]. Early antiviral treatment is crucial to alleviate disease progression and the desired endpoint of treatment is HBsAg seroclearance, referred to as “functional cure”, resulting in improved long-term prognosis [3]. Interferon has an advantage in HBsAg loss with a higher efficacy to clear HBsAg (3∼7% after 1 year) compared with nucleos(t)ide analogues (NAs) (0∼3% after 1 year) [1]. Moreover, the rate of HBsAg loss can be improved in patients who have optimal baseline characteristics (22.2% for baseline HBsAg levels < 1500 IU/ml) [4]. But it’s limited by high cost and adverse events [5], identifying appropriate patients who are more likely to clear HBsAg during the early treatment phase is essential. Predictive factors such as high baseline alanine aminotransferase (ALT), low baseline HBV DNA, HBV genotype, younger individuals, and females are all independent predictors of PEG-IFN response; however, their positive and negative predictive values are low [1]. Furthermore, most researches have focused on the changes of on-treatment potential biomarkers, lacking valuable pretreatment parameters to predict HBsAg loss.

MicroRNAs are endogenous small non-coding RNAs (19-23 nt) that regulate gene expression by degrading and suppressing the translation of target mRNAs. Evidences indicated that miRNAs were associated with immune response outcomes during CHB treatment [6] especially miRNAs enriched in immunocytes can predict the immune response to interferon treatment [7]. We wonder whether miRNAs in immunocytes can be used as potential predictors for HBsAg loss. Due to miRNAs from plasma/serum are derived from many tissues throughout the body and their levels could be affected by many factors, we finally chose peripheral blood mononuclear cells (PBMC) containing immune cells with relatively stable miRNAs. We focused on patients who have high rate of HBsAg loss with low baseline HBsAg levels and HBeAg negative to detect potential predictive biomarkers.

## Methods

### Study participants and sample preparation

Patients who visited the out-patient Department of Infectious Disease, Southwest Hospital, Third Military Medical University were selected for the prospective study. This study was registered in the Chinese Clinical Trial Register (ChiCTR-ROC-16008735). There were sixty-seven patients available in total that have finished the treatment and be followed up for a period of time in our study at the time point of our analysis. All patients received regular follow-ups and clinical examinations. They met the following criteria (1) age range between 18 and 65 years old; (2) HBsAg positive for more than 6 months, baseline HBsAg level < 1500 IU/ml, HBV DNA < 1000 IU/ml, HBeAg negative, ALT ≤2 ULN (the upper limit of normal); (3) interferon treatment naïve, NAs treated or naïve; (4) excluded due to human immunodeficiency virus, hepatitis C virus and hepatitis D virus coinfection; (5) excluded with liver cirrhosis, hepatocellular carcinoma, non-alcoholic steatohepatitis, et al.; (6) no treatment for immunological regulation within six months; (7) women who had no plans to be pregnant. All enrolled patients had their PBMC isolated and stored in liquid nitrogen prior to PEG-IFNα-2a treatment.

### Response estimation and treatment regimen

The treatment effect was determined by HBsAg clearance. Subjects who achieved HBsAg clearance (HBsAg < 0.05 IU/ml) or HBsAg seroconversion (HBsAg < 0.05 IU/ml, HBsAb > 10 IU/ml) at the time of drug discontinuation and follow-ups were regarded as responders and included in the HBsAg clearance group. Those in whom HBsAg was not cleared were classified into the non HBsAg clearance group.

Our study was a cohort study, subjects were divided into two cohorts: the identification cohort and the confirmation cohort. The identification cohort was conducted by using the matched case-control study, ten subjects acquiring HBsAg clearance earliest were considered as the case group and the other ten without HBsAg clearance were included into the control group according to the proportion of 1:1 with their key baseline characteristics matched with HBsAg clearance subjects, for example, baseline HBsAg level, HBV DNA, ALT, history of NAs and drugs combination. The rest subjects consisted of the confirmation cohort.

After being informed the benefits and risks of treatment, the enrolled subjected received the treatment. The final duration and regimens were decided by experienced clinicians based on the actual situations and treatment regimens were adjusted based on clinical symptoms but observed the following principal. They were treated with PEG-IFNα-2a (180 μg/w) with a planned course at least 48 weeks, in combination with or without NAs, but no more than 96 weeks and ended the treatment until acquiring HBsAg clearance or HBsAg level remaining stable for 12 weeks. Treatment duration was prolonged when HBsAg level was lower than 50 IU/ml at 48 weeks, but no more than 96 weeks. The treatment duration was permitted less than 48 weeks for subjects discontinuing drugs in advance, because HBsAg levels were not significantly declined or even increased. Subjects with treatment duration less than 24 weeks or those who were unwilling to complete the treatment and discontinued drugs ahead of schedule though HBsAg level decreased were removed in our study.

### Laboratory measurements

Plasma samples were quantified at every follow-up point during treatment and after drugs discontinuation every 12 weeks. Architect platform (Abbott laboratories, Chicago, USA) was used for HBV markers quantification, including HBsAg, anti-HBs, anti-HBe, HBeAg, anti-HBc. HBsAg was measured with a dynamic range of 0.05–250 IU/ml. Samples were diluted and measured again if they were higher than 250 IU/ml. HBsAg levels below 0.05 IU/ml was regarded as HBsAg clearance. HBsAb greater than 10 IU/ml was defined as HBsAg seroconversion. Serum HBV DNA load was quantified using Detection Kit for Quantification of Hepatitis B virus DNA (the LightCycler48 Real-time PCR instrument, Roche, Basel, Switzerland). The lower limit of HBV DNA detection was 500 IU/ml in our lab test, and some samples were verified through COBAS HBV DNA test (Roche, Basel, Switzerland, lower limit of detection is 20 IU/ml). White blood cell counts, liver and renal function, coagulation and thyroid function were supervised in case of severe adverse events to guide and adjust clinical treatment regimens.

### RNA extraction

The PBMC from venous blood was isolated by Ficoll (Lymphoprep™) and resuspended in Fetal Bovine Serum (HyClone™, Cat.N:SV30087.03) and stored in liquid nitrogen until RNA extraction. Total RNA was extracted using QIAGEN miReasy Mini kit (Cat No/ID: 217004) according to the manufacturer’s instruction. RNA quality and quantity was measured using the Nanodrop spectrophotometer (ND-1000, Nanodrop Technologies) and RNA integrity was determined by gel electrophoresis.

### MicroRNAs microarray

RNA labeling and array hybridization were conducted according to their Exiqon’s manual respectively. After quality control, total RNA (1μg) was labeled using the miRCURY™ Hy3™/Hy5™ Power labeling kit (Exiqon, Vedbaek, Denmark, Cat#208032-A) and hybridized using the miRCURYTM LNA Array (v.19.0) (Exiqon) according to the array manual. Slides were scanned using the Axon GenePix 4000B microarray scanner (Axon Instruments, Foster City, CA). Scanned images were then imported into GenePix Pro 6.0 software (Axon) for grid alignment and data extraction. Data were normalized using the median normalization and differentially expressed miRNAs were identified through fold change and *p*-values. Hierarchical clustering and volcano plots demonstrated differential miRNAs expression profiles among the samples.

Real-time quantitative reverse-transcription polymerase chain reaction for miRNAs.

The differential miRNAs were ranked according to their fold change. According to the selection criteria: fold change > 3, *p* value < 0.05, we chose the top twelve differentially expressed miRNAs and examined their expression levels by qRT-PCR. Among them, eight was upregulated in HBsAg clearance group and four was upregulated in non-HBsAg clearance group. The quantitative reaction was performed using the universal microRNA qPCR Quantitation kit (Cat#GMRS-001, Genepharma, Shanghai). All primers for qRT-PCR(shown in Table 1)were synthesized by GenePharma company, Shanghai. For cDNA synthesis and qRT-PCR, the reaction was performed according to the manufacture’s instruction. To normalize miRNA levels between samples, U6 small nuclear RNA was used as the internal control. The expression levels of the differential expressed miRNAs between the two groups were calculated using the ΔCT method (ΔCT = CT of interest miRNA-CT of internal control U6).

**Table 1:** primers sequence of miRNAs

Gene		Sequence
hU6	F primer	CAGCACATATACTAAAATTGGAACG
	R primer	ACGAATTTGCGTGTCATCC
hsa-miR-3960	F primer	ATATTGCGGCGAGTCCGA
	R primer	TATGGTTGTTCACGAGTCCTTGTC
hsa-miR-23a-3p	F primer	GAAGTCTATCACATTGCCAGGG
	R primer	TATGGTTGTTCTCGTCTCTGTGTC
hsa-miR-335-5p	F primer	CGTCCTCGTCAAGAGCAATAAC
	R primer	TATGCTTGTTCTCGTCTCTGTGTC
hsa-miR-126-3p	F primer	ACAGTTCTCTCGTACCGTGAGTAAT
	R primer	TATGGTTTTGACGACTGTGTGAT

Prediction of target genes of differential expressed miRNAs and GO/Pathway analysis.

The intersection of differential expressed miRNAs target genes were analyzed by TargetScan7.1 and miRDBV5.0. Predicted target genes of miRNAs (shown in Additional file 1) were then analyzed by GO and Pathway. GO contigs are divided into biological processes, cellular components and molecular functions according to GO terms. Pathway analysis is a functional analysis mapping genes to Kyoto Encyclopedia of Genes and Genomes (KEGG) pathways. Fisher’s exact test was used to determine significant GO terms and pathways.

### Statistical analysis

All statistical analyses were performed using the Statistical Package for the Social Sciences (SPSS, version23) and Stata Statistical Software (STATA, version13.1). Discrete data, normally distributed and skewed distribution continuous data were presented as counts and percentages, means ± SD and medians with the accompanying inter-quartile ranges (IQR) respectively. The differences between groups were analyzed using Chi-square or Fisher’s exact test, student-t tests and the Mann-Whitney U test. Univariate and multivariate logistic regression analyses (using stepwise method) were performed to evaluate the magnitude and significance of the association. The choice of models was under the simple and feasible evaluation criterion of the Akaike information criterion (AIC). ROC and AUC analysis was performed to determine the sensitivity and specificity of PEG-IFN response and optimal cut-off was selected based on Youden Index (YI). A two-sided *p* value< 0.05 was considered statistically significant.

## Results

### Baseline characteristics and treatment outcome of participants

There were 79 subjects with treatment duration over 24 weeks in total at the time point of our analysis, 12 subjects who were unwilling to complete the treatment were excluded that we didn’t know the actual response outcomes. In the end, a total of 67 subjects (55 males and 12 females) were available for the study and divided into two cohorts, the identification cohort (*n* = 20) and the confirmation cohort (*n* = 47). There were twenty-seven subjects achieving HBsAg clearance (27/67) and twenty-one subjects acquiring HBsAg seroconversion (21/67), nine in identification cohort and twelve in confirmation cohort. The baseline characteristics of the subjects for each cohort are presented in Table 2. Significant differences in HBsAg levels at the end of treatment (EOT) and follow-ups were observed in the two cohorts. There were no statistical differences in other baseline characteristics such as gender, HBV DNA and ALT levels. However, age in the identification cohort and baseline HBsAg levels in the confirmation cohort were statistical significant.

**Table 2:** Baseline characteristics and treatment outcome of two cohorts

Characteristic	Identification Cohort (*n* = 20)			Confirmation Cohort (*n* = 47)		
	HBsAg clearance	Non-HBsAg clearance	*p*	HBsAg clearance	Non-HBsAg clearance	*p*
No.	10	10	–	17	30	–
Male sex, No.(%)	9 (90%)	7 (70%)	0.582	15 (88%)	24 (80%)	0.692
^a^Age, y	29 ± 8.9	46.4 ± 6.8	0.0001^***^	39 ± 7.7	38.7 ± 9.3	0.921
History of NAs	–	–	–	–	–	–
Yes	6	7	1	10	22	0.305
No	4	3	–	7	8	–
Combination of NAs	–	–	–	–	–	–
Yes	4	4	1	5	8	0.84
No	6	6		12	22	
^c^HBV DNA, IU/ml	–	–	–	–	–	–
undetectable (< 500)	7	9	0.582	16	23	0.228
500~ 1000	3	1	–	1	7	–
^b^HBsAg at baseline, IU/ml	186.09 (29.31–724.35)	184.28 (62.75–536.69)	1	116.54 (33.36–195.26)	504.36 (156.35–875.49)	0.01
< 500, No.	7	7	0.549^$^	14	15	0.09^$^
500~ 1000, No.	2	3	–	2	10	–
1000~ 1500, No.	1	0	–	1	5	–
^a^ALT baseline level, IU/ml	36.4 ± 17.83	30.69 ± 9.9	0.388	31.51 ± 14.0	37.21 ± 30.65	0.474
^a^WBC,× 10^12^	6.12 ± 1.43	6.08 ± 1.37	0.951	5.98 ± 1.70	5.37 ± 1.28	0.176
Treatment outcome	–	–	–	–	–	–
^b^PEG-IFN treatment course, w	62.5 (49.8–70.8)	49.5 (39.3–59.5)	0.89	68 (48–72)	71 (48–72.75)	0.789
^b^HBsAg at EOT, IU/ml	0.01 (0–0.014)	228.3 (11.80–387.92)	1.53E-4^***^	0 (0–0.01)	20.86 (2.6–154.47)	1.5783E-8^***^
Follow-up weeks	56.3 (33.3–93.8)	45.6 (32.4–80)	0.241	25.6 (19.1–37.7)	23.6 (14.3–39.1)	0.542
^b^HBsAg at follow-up, IU/ml	0.015 (0–0.02)	184.26 (38.45–380.22)	1.44E-4^***^	0 (0–0.02)	28.11 (4.94–289.45)	1.637E-8^***^

^$^a` = (2*a)/[k*(k-1) + 1] = 0.014, k = 3, a = 0.05; * *p* < 0.05, **p < 0.01, ***p < 0.001

*ALT* alanine aminotransferase, *WBC* white blood count, *EOT* end of treatment. History of NAs means whether they have used nucles(t)ide analogs (NAs) before PEG-IFN treatment, the NAs include all kinds of conventional medicines: TDF, ETV, LAM, LdT, ADV. They were used by means of monotherapy or combined treatment. The median duration time of NAs was 64.60w (33.3–82.97) (median ± IQR**)**

^a^data was presented as mean ± SD

^b^data was presented as median (IQR range)

^c^HBV DNA < 500 IU/ml is undetected in our lab test

### Identification and validation by qPCR-PCR of candidate miRNAs

**Fig. 1:**
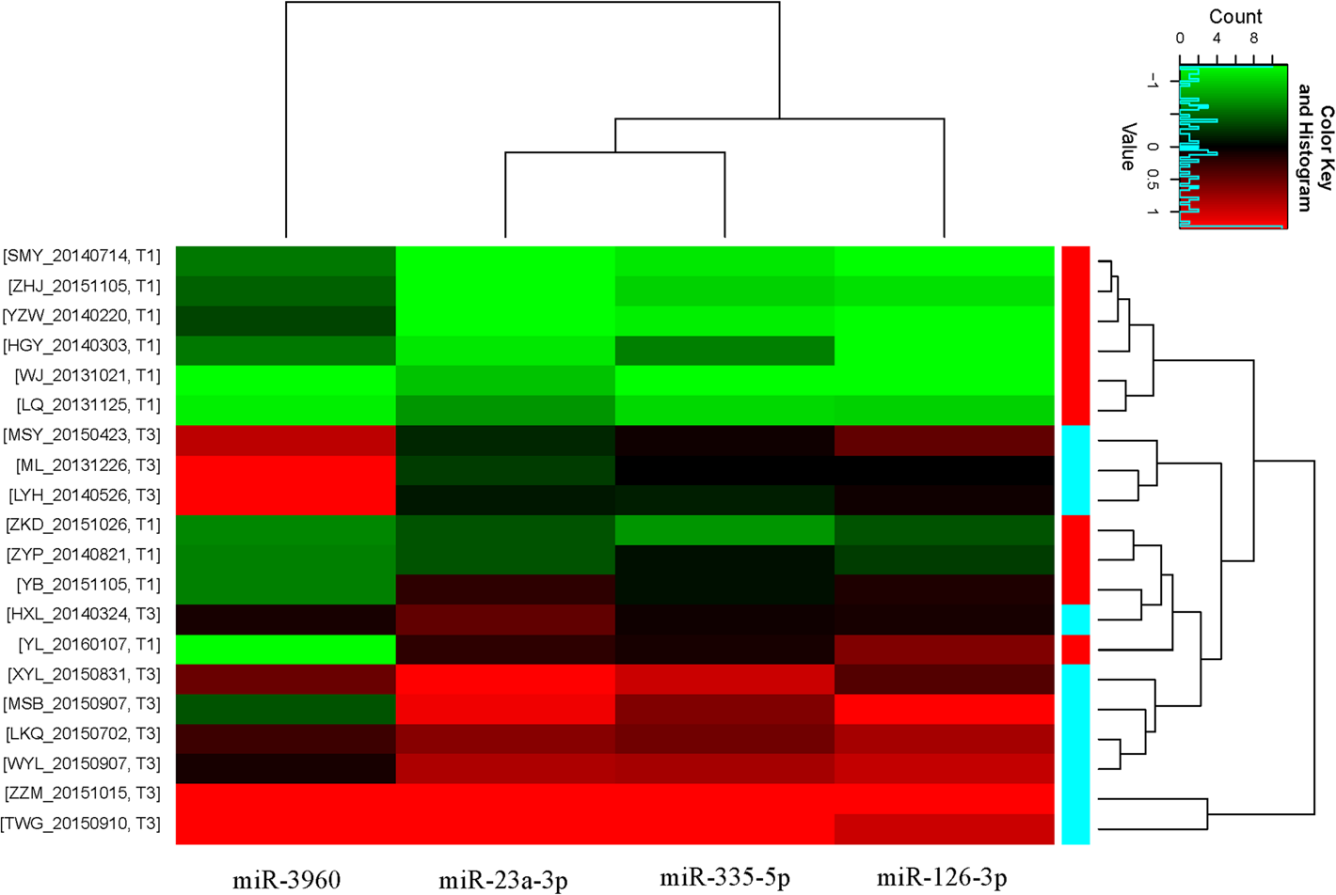
Hierarchical clustering of verified differentially expressed four miRNAs in the identification cohort. T1 and T3 stand for response group and non-response group respectively, the heatmap shows scaled expression values with highest values in red and lowest in green. SMY_20140714, ZHJ_20151105 et al. stands for the name and time of samples

**Fig. 2:**
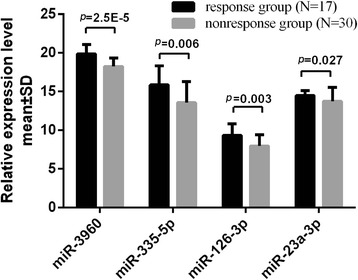
The relative expression levels of the differential miRNAs between the two groups in the confirmation cohort. The relative level is shown as ΔCT (ΔCT = CT of interest miRNA-CT of internal control U6, *p* < 0.05). Statistical significant is analyzed through student-t tests between the two different response groups. A two-sided *p* value < 0.05 was considered statistically significant

MicroRNA array (Exiqon v.7) was used to detect the differentially expressed miRNAs. The array consisted of 3100 capture probes, covering all human, mouse and rat miRNAs annotated in miRBase19.0. The differential expressed miRNAs were determined in the identification cohort that consisted of 10 patients from HBsAg-cleared (response group) and 10 non-HBsAg cleared (nonresponse group). Total of 417 miRNAs were found differential expressed (> 1.5-fold, *p* < 0.05): 342 upregulated and 75 downregulated (clustering and volcano plots are shown in Additional file 2: Figure S1 and Additional file 3: Figure S2). In order to validate the microarray results, the top twelve miRNAs were selected to validate the expression levels using qRT-PCR in the confirmation cohort (*n* = 47) according to the selection criteria: fold change > 3, *p* value < 0.05. Four out of twelve had statistical significant (*p* < 0.05) (miR-3960, miR-126-3p, miR-335-5p, miR-23a-3p). These four miRNAs levels were downregulated in the response group compared with the nonresponse group. Hierarchical clustering in the identification cohort and the relative expression levels in the confirmation cohort of the four miRNAs are shown in Figs. 1 and 2.

### Predictors of HBsAg clearance: Logistic regression analysis and ROC results

**Fig. 3:**
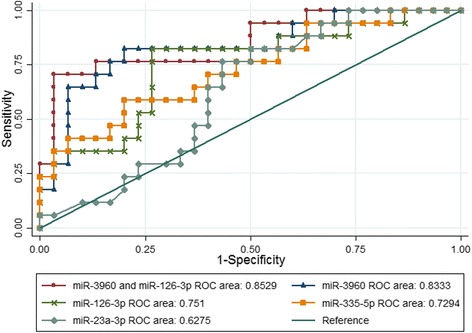
ROC curve. The receiver operating characteristic curve analysis for the predictive efficacy of the differentially expressed miRNAs for HBsAg clearance

To further evaluate the predictive capability of the PBMC miRNAs for HBsAg clearance, subsequent univariate/multivariate logistic regression and ROC analysis was performed and the results are shown in Tables 3 and 4. The results of the univariate logistic regression analysis demonstrated that lower levels of miR-3960 (*p* = 0.001), miR-126-3p (*p* = 0.01) and miR-335-5p (*p* = 0.013) were associated with HBsAg clearance and their AUCs for prediction were 0.8333 (*p* = 0.001) for miR-3960, 0.751 (*p* = 0.01) for miR-126-3p and 0.7294 (*p* = 0.013) for miR-335-5p (ROC curve shown in Fig. 3). We further conducted multivariate stepwise logistic regression analysis, miR-335-5p, miR-23a-3p and miR-126-3p had no statistical significant when were included in regression equation while miR-3960 remained statistical significant (*p* = 0.003). The combination of miR-3960 and miR-126-3p had higher specificity and PPV (96.67 and 92.31%, respectively), but its AUC had no significant difference compared with miR-3960 (0.856 vs. 0.833, *p* = 0.567). The best cut-off and OR value of miR-3960 ware 18.96 (ΔCT) and 3.354. It meant that the possibility of HBsAg clearance would increase with the rising ΔCT_miR-3960_. Taken together, miR-3960 may be an optimal predictive biomarker for HBsAg clearance.

**Table 3:** Univariate and multivariate logistic regression analysis for predicting HBsAg clearance

Variables	Univariate Logistic regression analysis					Multivariate Logistic regression analysis				
	*p*	B	OR	95% CI	AIC	*p*	B	OR	95% CI	AIC
miR-3960	0.001	1.210	3.354	1.638, 6.868	47.832	0.003	1.116	3.053	1.448, 6.436	45.028
miR-126-3p	0.01	0.794	2.213	1.213, 4.038	55.493	0.056	0.643	1.901	0.984, 3.672	
miR-335-5p	0.013	0.380	1.462	1.082, 1.976	57.139	–	–	–	–	–
miR-23a-3p	0.094	0.539	1.714	0.912, 3.223	61.399	–	–	–	–	–

*B* regression coefficient, *OR* odds ratio, *CI* confidence internal, *AIC* Akaike information criterion

**Table 4:** Receiver operating characteristic curve analysis for predicting HBsAg clearance

Variables	Specificity (%)	Sensitivity (%)	PPV (%)	NPV (%)	AUC	YI	Best Cut-off	*p*
miR-3960	93.33	64.71	84.62	82.35	0.8333	0.624	18.96	–
miR-126-3p	86.67	35.29	60.00	70.27	0.7510	0.557	8.64	–
miR-335-5p	90.00	41.18	70.00	72.97	0.7294	0.388	15.67	–
miR-23a-3p	83.33	11.76	28.57	62.50	0.6275	0.331	14.24	–
miR-3960 + miR-126-3p	96.67	70.59	92.31	85.29	0.8529	0.673	–	0.567^a^

*PPV* positive predict value, *NPV* negative predict value, *AUC* area under curve, *YI* Youden index

^a^compared with AUC of miR-3960

### Functional annotation of the target genes for miRNAs

To further identify the putative functions of the four identified miRNAs, their target genes were analyzed using Target7.1 and miRDBV5.0. Go/Pathway analysis was performed for the target genes that intersected for the individual miRNAs. Significantly enriched GO terms for each miRNA target genes included regulation of metabolic process, signal transduction, cell communication and others. In addition, KEGG analysis was conducted to elucidate the biological pathways of the differential expressed miRNAs target genes (shown in Additional file 4). The enriched categories of target gene pathways included, microRNAs in cancer, mTOR signaling pathway, NOD-like receptor signaling pathway, NF-κB signaling pathway and others. We hypothesize that these miRNAs possibly play a critical role in HBsAg clearance of CHB through the regulation of genes expression of signaling pathways associated with immune response.

## Discussion

The aim of our study is to find potential predictive biomarkers to select appropriate CHB patients to accept PEG-IFNα-2a treatment. In this study, we determined that four differentially expressed miRNAs were associated with HBsAg clearance before treatment. The four miRNAs levels were consistent with the microarray results and their levels were significant lower in the response group than in the nonresponse group. MiR-3960 (*p* = 0.001), miR-126-3p (*p* = 0.01) and miR-335-5p (*p* = 0.013) were all significant predictors (*p* < 0.05) using univariate logistic regression, having higher AUC and PPV than miR-23a-3p (*p* = 0.094). In order to establish the optimal predictive model, multivariate logistic regression analysis was conducted. Results showed that miR-3960 was the optimal predictive biomarker for HBsAg clearance which remained statistical difference in the multivariate logistic regression analysis. Though AUC and PPV of the combination of miR-3960 and miR-126-3p were higher than single miR-3960 (0.853 vs. 0.833 for AUC, 92.31% vs. 84.62% for PPV), there was no significant difference, which may be explained by the relative small sample size. Therefore, larger sample studies are needed to verify the predictive capability of combined biomarkers. MiR-3960 had ideal PPV of 84.62%, it meant that there was an accuracy of 84.62% to be correct when patients were predicted to have HBsAg clearance before treatment as determined by miR-3960 levels (when their ΔCT value of miR-3960 is higher than the cut-off 18.96). If CHB subjects were HBeAg negative, HBsAg < 1500 IU/ml and HBV DNA < 1000 IU/ml, we can evaluate whether subjects will acquire HBsAg clearance in advance by detecting pretreatment miR-3960 expression level, which can raise patients’ and physicians’ confidence in treatment. As determined by our literature review, we are the first to demonstrate that pretreatment PBMC miRNAs levels could be used to predict HBsAg loss in CHB patients with low baseline HBsAg levels.

Several studies have reported that patients in different phases of CHB had diverse expression profiles of miRNAs [8]. This may closely correlate with the different immune states of CHB. We know that miRNAs participate in the regulation of immune system [9]. HBsAg clearance is a complex process involving various factors and immunoregulation is considered as an indispensable factor. Patients who achieve HBsAg clearance may already be in a good immune condition before PEG-IFN treatment which may promote the beneficial effects of PEG-IFN by promoting interferon’s immune regulatory function. The differential expressed miRNAs may be associated with the initial immune state. We analyzed the biological significance of the identified miRNAs. MiR-3960 regulates the differentiation of monocyte/dendritic cell (DC) through competitively binding to long noncoding RNA HOTAIRMI [10]. DCs are professional antigen presenting cells that play an important role in antigen presentation and link the innate and adaptive immune systems. MiR-126-3p was reported to suppress tumor metastasis and angiogenesis of hepatocellular carcinomas by targeting LRP6 and PIK3R2, and its restoration may be a promising strategy for HCC therapy [11]. As for miR-335-5p [12] and miR-23a-3p [13], they are associated with the metastasis and prognosis of other tumors, such as osteosarcoma and prostate cancer. The immune system plays a major role in immune surveillance against tumors [14], and metastasis and invasion of tumors may be associated with dysfunction of the immune system. Hence differential expressed miRNAs may have an impact on the immune system and correlate with HBsAg clearance. To comprehensively investigate the molecular mechanisms of the four miRNAs associated with HBsAg clearance, we performed GO/Pathway enrichment analysis. The analysis showed that they regulate gene expression associated with multiple signal pathways. These pathways were important biological processes, such as inflammatory response, tumor progression and autophagy. For example, PIK3/Akt/mTOR signaling pathway was correlated with HBV replication [15] and it may be the result of IGF-1R/PIK3/Akt/mTOR signaling-induced autophagy [16]. NOD like receptor (a cytoplasmic pattern recognition receptor) can link the innate and adaptive immune system [17]. However, the underlying mechanism of the identified miRNAs associated with HBsAg clearance requires further study.

Our study demonstrated differential expression miRNAs in pretreatment PBMC can act as potential predictors for HBsAg clearance. They may contribute to HBsAg clearance that involve complex biological processes and require further study to demonstrate their roles. There were several limitations of our study. Firstly, the number in the confirmation cohort was small, a larger study will be helpful to verify our findings. Secondly, we only selected twelve miRNAs to verify our microarray results due to limited sample amounts, however the unselected miRNAs may also act as predictors for HBsAg loss. Thirdly, miRNAs expression levels were only measured at pretreatment, their changes during treatment and HBsAg clearance were unknown. It may help us to understand the relationship between miRNA and HBsAg clearance and find new predictive biomarkers. Hence a longitudinal study of miRNAs expression patterns needs to be performed. Finally, age has statistical difference in the two response groups of identification cohort, the reason was that we firstly balanced other important factors such as baseline HBsAg level, HBV DNA, ALT, et al. Whether age has an influence on response outcomes is still controversial with low negative and positive predictive values [1], so we considered age as the less important factors. Age in the confirmation cohort was indifferent between response and non-response groups, which demonstrated that age had little influence on miRNAs expression in our cohort and made our conclusion applicable in a larger age range.

## Conclusion

In summary, miR-3960, miR-126-3p and miR-335-5p were associated with HBsAg clearance. MiR-3960 was the optimal predictor with statistical difference in univariate and multivariate logistic regression analysis. But whether the three miRNAs as predictors for PEG-IFN treatment of CHB patients in clinical application still need further study considering the small sample size and the unknown molecular mechanism between HBsAg clearance and miRNAs. Larger cohort studies and mechanism studies are required to determine the application value of these identified miRNAs in HBsAg clearance.

## Additional files

Additional file 1: Predicted target genes of miRNAs (XLSX 37 kb)

Additional file 2: **Figure S1.** Hierarchical clustering of differentially expressed miRNAs in the identification cohort. T1 and T3 stand for response group and non-response group respectively, the heatmap shows scaled expression values with highest values in red and lowest in green. ML_20131226, LYH_20140526 et al. stands for the name and time of samples. (PNG 145 kb)

Additional file 3: **Figure S2.** Volcano plots for differential expressed miRNAs in the identification cohort. The vertical lines correspond to 1.5-fold up and down respectively, and the horizontal line represents a *p*-value of 0.05. The red point in the plot represents the differentially expressed miRNAs with statistical significant (PNG 65 kb)

Additional file 4: The biological pathways of the differential expressed miRNAs target genes (XLSX 24 kb)

## Abbreviations

GO: Gene ontology
KEGG: Kyoto Encyclopedia of Genes and Genomes
miRNA: microRNA
NAs: Nucleos(t)ide analogues
PBMC: Peripheral blood mononuclear cells
PEG-IFN: Pegylated interferon
qRT-PCR: Quantitative reverse-transcription polymerase chain reaction
