# Supplementary figures and images for: Pretreatment microRNA levels can predict HBsAg clearance in CHB patients treated with pegylated interferon α-2a
Source: Virol J. 2018 Apr 23;15:73. doi: 10.1186/s12985-018-0982-y (PMC5914056; doi:10.1186/s12985-018-0982-y)

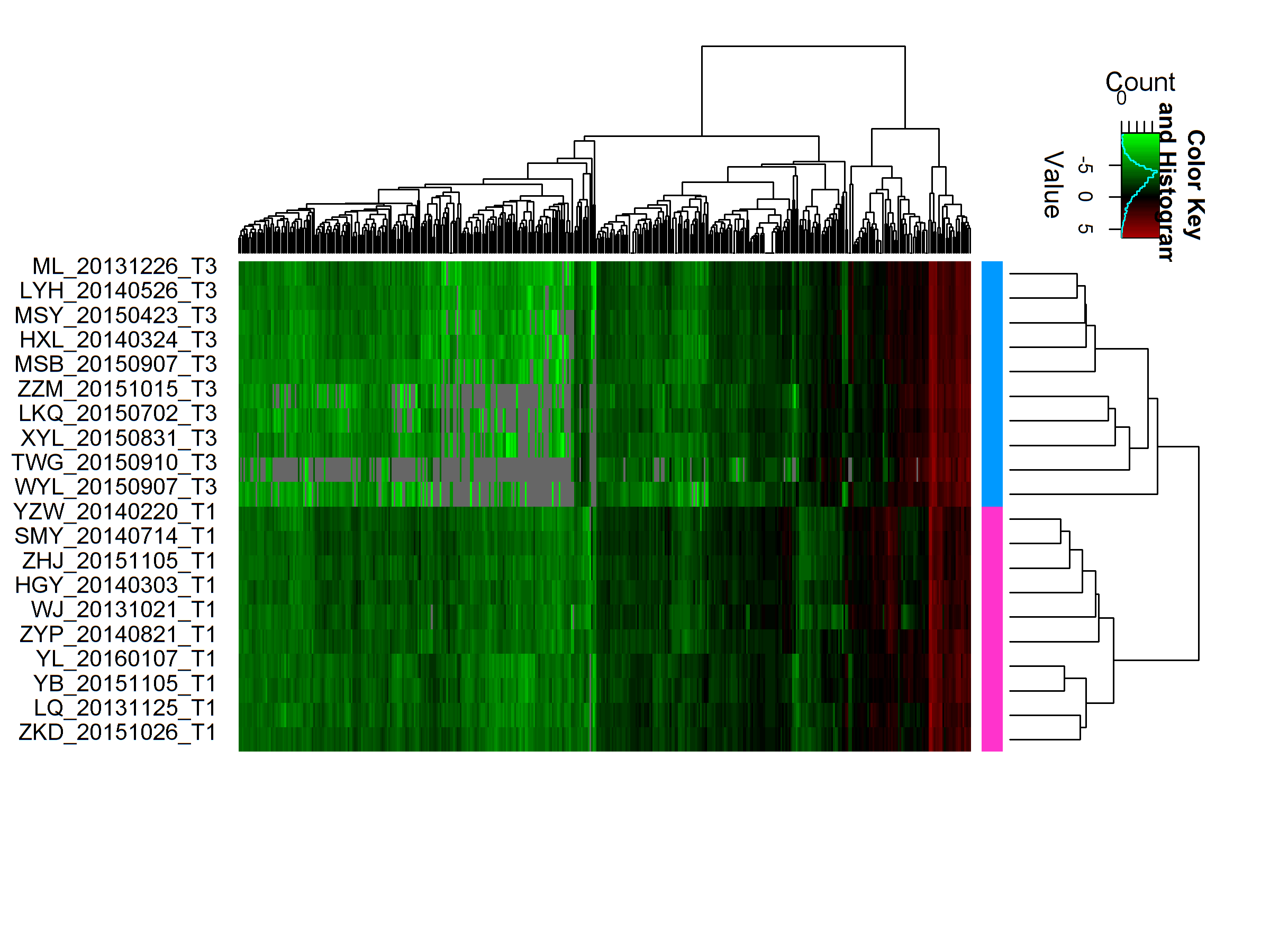

Supplement: Supplementary file 2 — Figure S1. Hierarchical clustering of differentially expressed miRNAs in the identification cohort. T1 and T3 stand for response group and non-response group respectively, the heatmap shows scaled expression values with highest values in red and lowest in green. ML_20131226, LYH_20140526 et al. stands for the name and time of samples. (PNG 145 kb) [file 12985_2018_982_MOESM2_ESM.png]

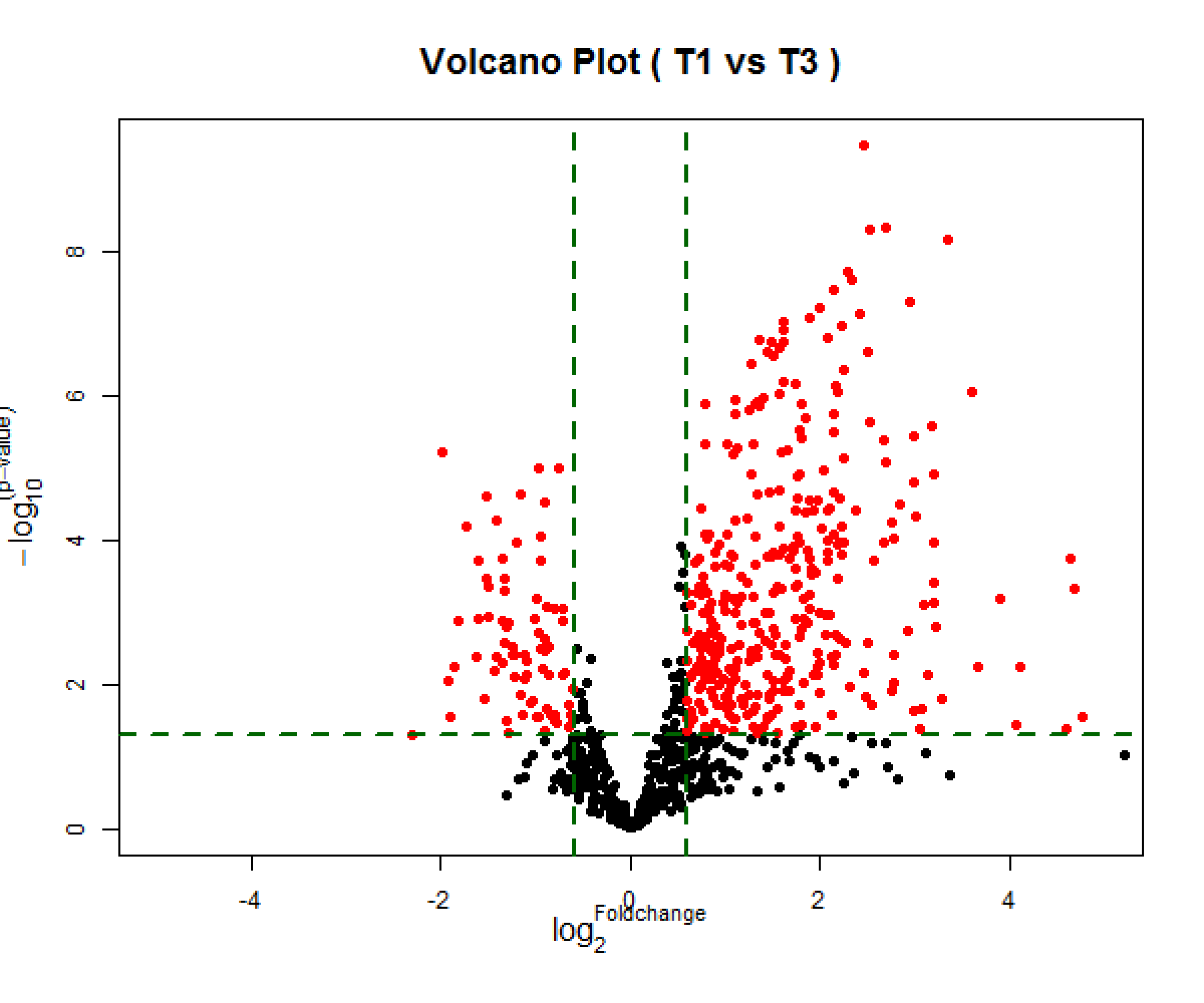

Supplement: Supplementary file 3 — Figure S2. Volcano plots for differential expressed miRNAs in the identification cohort. The vertical lines correspond to 1.5-fold up and down respectively, and the horizontal line represents a p-value of 0.05. The red point in the plot represents the differentially expressed miRNAs with statistical significant (PNG 65 kb) [file 12985_2018_982_MOESM3_ESM.png]
